# Inter-and intraspecific variation in fern mating systems after long-distance colonization: the importance of selfing

**DOI:** 10.1186/1471-2229-12-3

**Published:** 2012-01-04

**Authors:** G Arjen de Groot, Betty Verduyn, ER Jasper Wubs, Roy HJ Erkens, Heinjo J During

**Affiliations:** 1 Ecology and Biodiversity group, Institute of Environmental Biology, Utrecht University, Padualaan 8, 3584 CH Utrecht, The Netherlands

## Abstract

**Background:**

Previous studies on the reproductive biology of ferns showed that mating strategies vary among species, and that polyploid species often show higher capacity for self-fertilization than diploid species. However, the amount of intraspecific variation in mating strategy and selfing capacity has only been assessed for a few species. Yet, such variation may have important consequences during colonization, as the establishment of any selfing genotypes may be favoured after long-distance dispersal (an idea known as Baker's law).

**Results:**

We examined intra-and interspecific variation in potential for self-fertilization among four rare fern species, of which two were diploids and two were tetraploids: *Asplenium scolopendrium *(2n), *Asplenium trichomanes *subsp. *quadrivalens *(4n), *Polystichum setiferum *(2n) and *Polystichum aculeatum *(4n). Sporophyte production was tested at different levels of inbreeding, by culturing gametophytes in isolation, as well as in paired cultures with a genetically different gametophyte. We tested gametophytes derived from various genetically different sporophytes from populations in a recently planted forest colonized through long-distance dispersal (Kuinderbos, the Netherlands), as well as from older, less disjunct populations.

Sporophyte production in isolation was high for Kuinderbos genotypes of all four species. Selfing capacity did not differ significantly between diploids and polyploids, nor between species in general. Rather selfing capacity differed between genotypes within species. Intraspecific variation in mating system was found in all four species. In two species one genotype from the Kuinderbos showed enhanced sporophyte production in paired cultures. For the other species, including a renowned out crosser, selfing capacity was consistently high.

**Conclusions:**

Our results for four different species suggest that intraspecific variation in mating system may be common, at least among temperate calcicole ferns, and that genotypes with high selfing capacity may be present among polyploid as well as diploid ferns. The surprisingly high selfing capacity of all genotypes obtained from the Kuinderbos populations might be due to the isolated position of these populations. These populations may have established through single-spore colonization, which is only possible for genotypes capable of self-fertilization. Our results therewith support the idea that selection for selfing genotypes may occur during long-distance colonization, even in normally outcrossing, diploid ferns.

## Background

In spite of its relatively infrequent occurrence, long distance colonization is of disproportionate importance to species range expansions [e.g. [1,2]]. Long-distance colonization requires plant species' to possess a distinct set of capabilities, not only related to the dispersal of propagules, but also to plant and population establishment upon arrival. This involves diaspore characteristics, plant ontogenetic and morphological traits, as well as reproductive strategies. Genotypes possessing these capabilities will have a selective advantage over other genotypes when colonizing new and distant habitats. This advantage is becoming more important in a world increasingly under the pressure of climate change and fragmentation of natural habitats [3].

Various studies on plants and animals have shown that individuals with higher dispersal capacities tend to be found with greater frequency towards species' range limits [4,5] and that these enhanced capacities tend to have a genetic basis [6]. Likewise, inbreeding rates often increase towards range margins [7]. This might partly be due to genetic isolation and small population sizes [8], but can also be explained by reproductive assurance [9]. As colonization of vacant patches near a species' range limits will often depend on rare events of diaspore arrival through long-distance dispersal [e.g. [2]], mate limitation is likely high [10] and colonization success may strongly depend on self-fertilization. For this reason, Baker [11,12] suggested that establishment of selfing individuals will be strongly favoured after long-distance dispersal. Baker's law [13] states that long-distance colonization may therefore result in selection for individuals with high self-fertilization potential. As a result, plants in young populations near a species' range limit sometimes show relatively low self-incompatibility [5]. However, whether such selection occurs and how long this effect remains visible in the populations after initial colonization, depends on the dominant mating strategy, as well as the intraspecific variation in mating strategy present in the species investigated [e.g. [14]]. Selection for genotypes capable of self-fertilization will not occur in species that lack any intraspecific variation in mating strategy. Moreover, the overrepresentation of selfing genotypes may be reduced with time since colonization as a result of inbreeding depression [14]: the reduced success of inbred progeny due to the expression of genetic load (i.e. recessive deleterious alleles).

In ferns, which alternate between two free-living generations (gametophyte and sporophyte), sexual reproduction takes place on the gametophyte. After a spore has reached a suitable habitat patch and has germinated, fertilization of the gametophyte is required for sporophyte establishment [15]. In homosporous ferns, gametophytes have the potential to become male, female or bisexual. Sexual status typically varies between individuals and depends both on genetic factors and environmental conditions [16]. The possibility of producing hermaphroditic gametophytes allows for self-fertilization of a single gametophyte (i.e. intragametophytic selfing [17]). This potential is of particular importance for fern colonization, as very limited gamete dispersal distances result in strong mate limitation. This may strongly limit options for cross-fertilization as long as no local spore sources are present [18,19]. Long-distance colonization thus might be primarily dependent on reproduction via single spores, through intragametophytic selfing [18]. This type of reproduction represents an extreme case of inbreeding. Fern gametophytes contain only half the number of sets of chromosomes of the somatic cells of the sporophyte. As gametophytes of diploid ferns thus have only a single copy of each chromosome, intragametophytic selfing in diploid ferns produces sporophytes that are homozygous at all loci. This results in the direct expression of any recessive deleterious alleles in the sporophytes produced, which may severely affect the fitness of inbred sporophytes [20]. Gametophytes of polyploids possess multiple copies of each gene. Therefore, both the gametophytes and the gametes they produce may contain multiple alleles per locus. Even after intragametophytic selfing, recessive deleterious alleles may therefore be masked by other alleles in the sporophyte, making the effects of inbreeding depression less pronounced. For that reason, polyploid ferns are generally assumed to show enhanced survival of inbred progeny, and higher population inbreeding rates [21].

Fern mating systems can be studied experimentally using breeding experiments [17]. Such experiments compare sporophyte production by obligate *intra*gametophytic selfing on isolated gametophytes with sporophyte production in paired cultures, in which case also *inter*gametophytic crossing is possible (or intergametophytic selfing, if the second gametophyte originates from the same parent sporophyte [20]). Ferns generally seem to lack genetic self-incompatibility mechanisms [22], but unsuccessful self-fertilization may be caused by a failure of the gametophyte to become bisexual, unsuccessful transport of spermatozoids to the female reproductive organs, or the presence of genetic load. Species differences in sex ratios, gametophyte morphology and genetic load may therefore result in different types of mating strategies. Together with studies on observed genetic variation in fern populations, past breeding experiments suggested that the mating strategies employed by fern species vary in a bimodal way: some species reproduce mainly by self-fertilization and others rely on obligate intergametophytic crossing [17,23-26]. However, some species do show mixed mating systems [27,28], and by now several studies have indicated that mating systems may vary greatly even between different genotypes within species [18,29,30]. This intraspecific variation is in line with the large variation in inbreeding rates observed among sites in population genetic studies [e.g. [31,32]]. However, as breeding experiments with multiple genotypes are very laborious, intraspecific variation in mating strategy has only been assessed for a few species. Due to this lack of data, it remains largely unknown to what extent selfing genotypes are also present in species previously described as typical outcrossers, and how intraspecific variation in mating strategy differs between diploid and polyploid species. Therefore, it is also unclear to what extent selection for selfing genotypes, sensu Baker [11,12], is a widespread phenomenon in ferns.

In this study, we simultaneously investigated inter-and intraspecific variation in mating strategy in four temperate fern species, including two diploid and two allotetraploid species. We performed breeding experiments on several genotypes per species, determining the success of sporophyte production at different levels of inbreeding. In this way, we tested whether intraspecific variation in mating system varied between species, whether genotypes with a high capacity to self-fertilize are present in all four species, and whether selfing capacities and overall mating strategies differed between diploid and polyploid species.

Most genotypes used were derived from young populations in the Kuinderbos, a planted forest on Dutch polder land recently reclaimed from the sea. As the four investigated species are all rare in the Netherlands, with nearest source populations located 30-350 km away, the populations in the Kuinderbos must have established following long-distance dispersal [33,34]. A population genetic study (G.A. de Groot, unpublished results) suggested that most populations are the result of independent colonization events. Because such populations have likely established from single spores [18], we predicted that the sampled genotypes might have relatively high capacity for self-fertilization. This capacity was, however, expected to be lower for the diploid than for the polyploid species. We found surprisingly high selfing capacities for all Kuinderbos genotypes of all four species, both compared to results for a few additional genotypes from less isolated populations and compared to results from previous studies on the same species. Here, we interpret our results in the light of Baker's law, and suggest that selection for selfing genotypes may occur across fern species with different ploidy levels.

## Methods

### Study species and sampling strategy

Four calcicolous fern species were selected that had colonized various sites in the Kuinderbos, but varied considerably in population size and minimal required dispersal distance prior to colonization. *Polystichum setiferum *(Forssk.) Moore ex Woynar is a diploid species, with only a few colonization sites in the Kuinderbos. This forest lies at the northern edge of its distributional range and the nearest source population was located 250 km away at the time of colonization [34]. The allotetraploid *Polystichum aculeatum *(L.) Roth has much more colonization sites in the forest, but must have dispersed at least 100 km before local arrival (Bremer, 2007). The other two species, *Asplenium scolopendrium *L. (diploid) and *Asplenium trichomanes *subsp. *quadrivalens *D.E. Meyer (allotetraploid), had source populations closer to the Kuinderbos. Nevertheless, their spores must have covered > 30 km to reach it [34]. Locally, *A. scolopendrium *is by far the most abundant of the rare fern species with numerous colonization sites in the forest. *Asplenium trichomanes *subsp. *quadrivalens *is only present at three sites with about ten sporophytes each.

In July 2008, spores were collected from plants at three sites per species in the Kuinderbos, which most likely represent different colonization events, as a population genetic study (G.A. de Groot, unpublished results) showed these populations to represent different gene pools. Entire fertile fronds were harvested of one plant per site. Additionally, spores were collected from one or more populations at > 200 km distance from the Kuinderbos. For the polystichoids, additional spores were collected from a plant obtained from a commercial grower. As some of the collected fronds did not contain enough spores for the experiment, some of the sampled sporophytes eventually could not be used for the experiment. An overview of all used plants, as well as their origin and applied code names, is presented in Table 1.

**Table 1:** Code and origin of parent plants used to obtain spores.

Species	Plant	Focus^4^	Locality	Habitat type	No. of ind.^5^
**ASPS**	AS1^1^	F	Kuinderbos, Flevoland, The Netherlands, site 1	Soil, on trench slope	206
	AS2^2^	F	Kuinderbos, Flevoland, The Netherlands, site 2	Soil, on trench slope	328
	AS3^3^	F	Kuinderbos, Flevoland, The Netherlands, site 3	Soil, on trench slope	262
	
	RC	NF	Rue de Caster, Liège, Belgium	Calcareous soil	> 200

**ASPT**	AT1	F	Kuinderbos, Flevoland, The Netherlands, site 1	Soil, on trench slope	9
	AT2	F	Kuinderbos, Flevoland, The Netherlands, site 2	Soil, on trench slope	9
	Eck	F	Eckelrade, Limburg, The Netherlands	Old garden wall	> 50
	
	B	NF	Bromney Common, London, UK	Old church wall	> 100

**POLS**	PS1	F	Kuinderbos, Flevoland, The Netherlands, site 1	Soil, on trench slope	15
	PS2	F	Kuinderbos, Flevoland, The Netherlands, site 2	Soil, on trench slope	110
	
	BRS	F/NF	obtained from Henk Braam B.V., fern grower	unknown	?

**POLA**	PA1	F	Kuinderbos, Flevoland, The Netherlands, site 1	Soil, on trench slope	31
	PA2	F	Kuinderbos, Flevoland, The Netherlands, site 2	Soil, on trench slope	112
	PA3	F	Kuinderbos, Flevoland, The Netherlands, site 3	Soil, on trench slope	74
	SG	F	Schone Grub, Limburg, The Netherlands	Calcareous soil	10
	
	BRA	NF	obtained from Henk Braam B.V., fern grower	unknown	?

ASPS = *Asplenium scolopendrium*, ASPT = *Asplenium trichomanes *subsp. *quadrivalens*, POLS = *Polystichum setiferum*, POLA = Polystichum aculeatum

^1^The same site was also sampled by Wubs et al. [30], but plants differ^2^Wubs et al. [30] used the same parent plant in their study, with code KB-4

^3^Wubs et al. [30] used the same parent plant in their study, with code KB-12

^4 ^F: gametophytes used as focal individuals (see text); NF: gametophytes used as non-focal individuals on paired dishes (inbreeding level II)

^5^The number of individuals present at the site where the plant was collected

### Genetic analysis

The collected sporophytes of each species were analysed for genotypic variation, by assessing allelic variation at four (species-specific) microsatellite loci per species, using previously published markers [35]. DNA was extracted from fresh leaf fragments using the GenElute™ Plant Genomic DNA Miniprep Kit (Sigma-Aldrich, St. Louis, USA). PCR amplification was performed using the primer sets and protocol described by De Groot et al. [35], fragment sizes were determined by automated detection using an ABI 3730 capillary sequencer (Life Technologies, Carlsbad, USA), and final allele scoring was performed using the ABI PeakScanner v.1.0 software.

The resulting genotypic data were primarily used to check if the plants used indeed represented different genotypes. Additionally, the data gave an indication of the homozygosity of the sporophytes. This has important consequences for the interpretation of the results, as sib gametophytes originating from a homozygous parent will be genetically identical. However, while inferring homozygosity is straightforward for the diploid species, this is more difficult for allotetraploids. Allotetraploid sporophytes originating from intragametophytic selfing will be homozygous at *homologous *chromosomes (originating from the same progenitor species), but can still contain variation at *homoeologous *chromosomes (originating from different progenitors). In ferns, which most often show a diploid pattern of inheritance [36], such individuals will show two alleles per locus, but their gametophytic offspring will all be genetically identical. However, genetic variation among sib gametophytes will surely exist if the parent plant shows more than two alleles per locus.

Throughout the rest of this text, the term 'genotype' is used to refer to the parental (sporophytic) genotypes and the genotypes of founding individuals. When discussing comparisons between replicate gametophytes we explicitly use the term 'gametophytic genotype'.

### Experimental design

Fronds were air-dried and, to get stock cultures of gametophytes, spores from each parental sporophyte were sown onto separate Petri dishes containing an autoclaved medium consisting of Parker's macronutrients and Thompson's micronutrients [37], solidified with 5.0 g L^-1 ^Gelrite^®^. Dishes were sealed with Parafilm^® ^and placed in a growth cabinet at 20°C, with a photoperiod of 16:8 h (light:dark) and 134.8 (± 8.3) μmol m^-2 ^s^-1 ^of photosynthetically active radiation (PAR). After 6 weeks, gametophytes had reached sufficient size to be transplanted to the experimental setting.

For each species, parental sporophytes from Kuinderbos sites and (when available) one distant population were indicated as focal plants. One sporophyte from a distant location was chosen as a non-focal plant. Gametophytes of focal plants were selected for experimental crosses on Petri dishes (Ø 6 cm) containing the same medium as described above. To compare reproductive success at different levels of inbreeding, two different treatments were used: gametophytes were either grown in isolation (treatment I) or paired with a gametophyte of a different genotype (from a spore of a non-focal plant) which was placed at 1 cm distance on the dish (treatment II). Each treatment was repeated 30 times for each of the focal sporophytes. All Petri dishes were randomly divided in groups of 18 dishes, which were then placed on the bottom of transparent plastic boxes of 20 × 30 cm, sealed with Parafilm^®^. These boxes were placed in a growth cabinet, under the same conditions as the stock cultures. For 30 weeks, all gametophytes of focal plants were examined for sporophyte production every two weeks. At the same time, all gametophytes were watered using a sterile pipette and sterile water to facilitate the movement of male gametes on and between gametophytes. For *P. setiferum*, Kuinderbos genotypes were crossed with the genotype from outside the forest in treatment II. However, sporophyte production of this non-focal plant was also recorded, in the paired setting as well as in additional isolated gametophytes, so that it could be interpreted as an additional focal genotype.

As we were interested in the overall capacity of the individual genotypes to produce sporophytes in isolation and in the presence of a second genotype, we did not select bisexual gametophytes, but transplanted gametophytes to the experimental setting just before they started to produce sexual organs. All transplanted gametophytes reached sexual maturity, as archegonia were observed on all individuals across species, genotypes and treatments. Whether these individuals were female or bisexual was however not assessed, as proper detection of antheridia proved very difficult without disturbing the gametophytes. Antheridia were however produced in the stock cultures of the genotypes involved.

### Data analysis

Using binary logistic regression analyses (SPSS, version 16.0, SPSS Inc., Chicago, USA), we assessed the effects of species, parental genotype and inbreeding level, as well as their interactions, on sporophyte production. First an overall analysis was performed on data from three time points (week 16, 22 and 30), including species, parental genotype (nested in species) and treatment as factors. Based on data from week 30, similar analyses were then performed separately per species, using parental genotype and treatment as factors.

In line with the definitions of Peck et al. [18] for measures of reproductive potential used in fern breeding studies, we define "gametophyte isolate potential" as the percentage of isolated gametophytes (thus replicates in treatment I) that produced a sporophyte. Therefore, in a second type of analysis, we tested for variation in gametophyte isolate potential among species of different ploidy level by excluding the data of treatment II and performing binary logistic regression analyses (again for week 16, 22 and 30) using ploidy level, species (nested in ploidy) and parental genotype (nested in species and ploidy) as factors. Again, we also performed separate tests of genotypic differences in gametophyte isolate potential per species.

As selection for selfing genotypes might potentially alter the effect of ploidy level and the amount of intraspecific variation in mating system specifically for isolated population, all analyses were repeated while excluding the genotypes from outside the Kuinderbos. Gametophyte mortality was monitored during the whole experiment. This information was used to calculate for each gametophyte the number of weeks that the gametophyte had been alive since transplantation. This 'realized fertilization time' [30] was incorporated as a covariate in all tests to correct for differences between gametophytes in opportunities for fertilization due to mortality.

## Results

### Genetic analysis

Each of the sporophytes used as a focal or non-focal plant in the experiment had a unique genotype (see Additional file 1). Within each species, parent sporophytes differed at a minimum of one of the four loci (plants AS1 and AS2 of *A. scolopendrium*; PS1 and PS2 of *P. setiferum*) and occasionally differed at all four loci (plants AT2 and Eck, as well as Eck and B of *A. trichomanes *subsp. *quadrivalens*). Kuinderbos genotypes of the two diploid species were homozygous at all tested loci, except for plant AS3. For the Kuinderbos genotypes of *A. scolopendrium *this was in line with previous results based on isozymes [30]. Genotypes from outside the Kuinderbos were heterozygous for both diploid species. Each tetraploid sporophytes was monomorphic for at least half the loci that were tested, and none of them showed more than two alleles per locus. These results support a homozygous status for the tetraploid plants, although heterozygosity cannot be excluded.

### Breeding experiments

Gametophyte mortality was very low for all species. Mortality rates started to increase after 22 weeks. Mortality at the end of the experiment (week 30) was highest in *Asplenium trichomanes *subsp. *quadrivalens *(20%; see Additional file 2).

**Figure 1:**
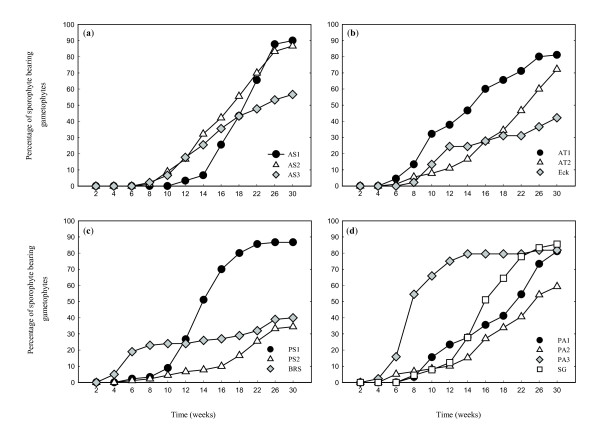
**Total sporophyte production across treatments, for each focal genotype**. Different panels show different species: **a**) *Asplenium scolopendrium*, **b**) *Asplenium trichomanes *subsp. *quadrivalens*, **c**) *Polystichum setiferum*, **d**) *Polystichum aculeatum*. For codes of genotypes see Table 1.

Although all gametophytes were transplanted six weeks after sowing, gametophytes of the *Asplenium *species were smaller than those of *Polystichum *and maturated slightly later. As a result, the first sporophytes were observed four weeks after transplantation for both polystichoids, while sporophytes of *A. trichomanes *subsp. *quadrivalens *and *A. scolopendrium *were not observed until six and eight weeks after transplantation, respectively (Figure 1).

Total sporophyte production of focal gametophytes was generally high across treatments in all species. From week 16 onwards, species differed significantly in overall production (Table 2), with highest mean production after 30 weeks in *A. scolopendrium *and *P. aculeatum *(78%) and lowest in *P. setiferum *(59%). Parental genotypes showed strong and significant differences in total sporophyte production in all species (Figure 1, Table 3). Genotypic differences were significant for all species in week 30. Particularly large variation was found among the two Kuinderbos genotypes of *P. setiferum*, with gametophytes of plant PS1 producing over 2.5 times more sporophytes than those of plant PS2 (Figure 1; Wald *χ*^2 ^= 41.043, *P *< 0.001).

**Table 2:** Separate binary logistic regression analyses for data from three time points during the experiment

Dependent factor	Independent factor	Statistic	Week 16^1^	Week 22^1^	Week 30^1^
**Total sporophyte**	Species	*Wald χ^2^*	14.973	14.299	32.610
**production**		*P*	**0.002**	**0.003**	**< 0.001**
		*df*	3	3	3
	
	Parental genotype	*Wald χ^2^*	94.964	106.080	105.704
		*P*	**< 0.001**	**< 0.001**	**< 0.001**
		*df*	9	9	9
	
	Treatment	*Wald χ^2^*	1.596	0.014	0.000
		*P*	0.206	0.906	1.000
		*df*	1	1	1
	
	Mortality	*Wald χ^2^*	6.389	13.140	3.204
		*P*	**0.011**	**< 0.001**	0.073
		*df*	1	1	1
	
	Species × treatment	*Wald χ^2^*	6.247	0.766	0.666
		*P*	0.100	0.858	0.881
		*df*	3	3	3
	
	Genotype × treatment	*Wald χ^2^*	37.296	32.832	28.138
		*P*	**< 0.001**	**< 0.001**	**0.001**
		*df*	9	9	9

**Gametophyte**	Ploidy level	*Wald χ^2^*	10.011	2.032	1.380
**Isolate potential**		*P*	**0.002**	0.154	0.240
		*df*	1	1	1
	
	Species	*Wald χ^2^*	5.521	11.440	24.173
		*P*	0.063	**0.003**	**< 0.001**
		*df*	2	2	2
	
	Parental genotype	*Wald χ^2^*	100.042	100.133	111.991
		*P*	**< 0.001**	**< 0.001**	**< 0.001**
		*df*	9	9	9
	
	Mortality	*Wald χ^2^*	3.924	10.816	0.026
		*P*	**0.048**	**0.001**	0.873
		*df*	1	1	1

^1^Significant effects (*P *< 0.05) appear in bold case

**Table 3:** Separate binary logistic regression analyses per species.

Dependent factor	Independent factor	Statistic	ASPS^1^	ASPT^1^	POLS^1^	POLA^1^
**Total sporophyte**	Parental genotype	*Wald χ^2^*	17.759	28.282	52.186	7.983
**production**		*P*	**< 0.001**	**< 0.001**	**< 0.001**	**< 0.001**
		*df*	2	2	2	3
	
	Treatment	*Wald χ^2^*	0.190	0.113	1.401	0.000
		*P*	0.663	0.737	0.237	0.998
		*df*	1	1	1	1
	
	Genotype × treatment	*Wald χ^2^*	10.029	0.179	17.564	0.481
		*P*	**0.007**	0.914	**< 0.001**	0.923
		*df*	2	2	2	3
	
	Mortality	*Wald χ^2^*	3.794	3.149	7.287	4.437
		*P*	0.051	0.076	**0.007**	0.035
		*df*	1	1	1	1

**Gametophyte**	Parental genotype	*Wald χ^2^*	56.905	32.341	126.113	7.034
**Isolate potential**		*P*	**< 0.001**	**< 0.001**	**< 0.001**	0.071
		*df*	2	2	2	3
	
	Mortality	*Wald χ^2^*	2.201	5.000	4.698	0.955
		*P*	0.138	**0.025**	**0.030**	0.328
		*df*	1	1	1	1

ASPS = *Asplenium scolopendrium*, ASPT = *Asplenium trichomanes *subsp. *quadrivalens*, POLS = *Polystichum setiferum*, POLA = Polystichum aculeatum

^1^Significant effects (*P *< 0.05) appear in bold case

**Figure 2:**
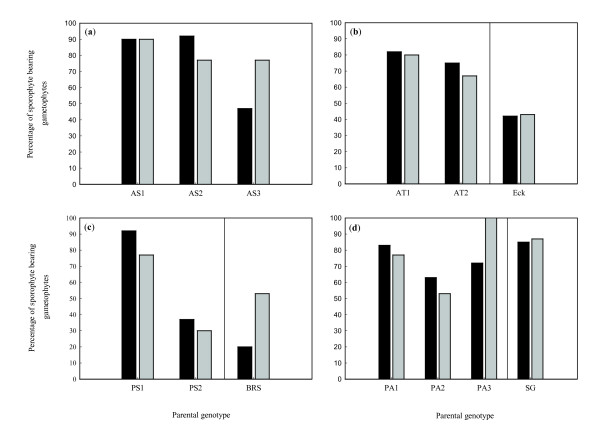
**Sporophyte production 30 weeks after transplantation, for all focal genotypes in each of the two treatments**. Black bars = I, isolated gametophytes (intragametophytic selfing); grey bars = II, among-population crossing (and intragametophytic selfing). Different panels show different species: **a**) *Asplenium scolopendrium*, **b**) *Asplenium trichomanes *subsp. *quadrivalens*, **c**) *Polystichum setiferum*, **d**) *Polystichum aculeatum*. For clarity a vertical line separates Kuinderbos genotypes (to the left, see Table 1 for codes of genotypes) and mainland genotypes (to the right).

Gametophyte isolate potential (percentage fertilization success at treatment I) was very high among the Kuinderbos genotypes for all four species (Figure 2). In each species we observed one or more parental genotypes of which a large majority of the isolated gametophytes (80-90%) produced sporophytes. Aborted zygotes were rarely observed in the experiment (single observation for each of the *Polystichum *species, none in *Asplenium*). Separate regression analyses including only the data of the isolated cultures showed significant differences in gametophyte isolate potential between ploidy level in week 16, but not in week 22 and 30 (Table 2). Gametophyte isolate potential significantly differed between species and parental genotypes. When tested separately per species, differences in gametophyte isolate potential between parental genotypes were not significant for *P. aculeatum *(*P *= 0.071) but very significant for the other species (Table 3). When parental genotypes from outside the Kuinderbos were excluded from the analysis, gametophyte isolate potential did not significantly differ between ploidy levels (Wald *χ*^2 ^= 0.182, *P *= 0.670), nor between species (Wald *χ*^2 ^= 3.283, *P *= 0.194). However, genotypic differences remained significant (Wald *χ*^2 ^= 71.487, *P *< 0.001).

For some genotypes fertilization success of focal gametophytes was higher when gametophytes were paired with another genotype (treatment II) than when gametophytes were cultured in isolation (treatment I). In none of the species, however, such an enhanced fertilization success on paired cultures was consistently observed among all parental genotypes tested. For *A. trichomanes *subsp. *quadrivalens *sporophyte production after 30 weeks was about equal among treatments in all genotypes (Figure 2). For the other three species, the overall pattern (across genotypes from all locations) was blurred by clear genotypic differences in the treatment effect. Both in *A. scolopendrium *and *P. aculeatum*, one Kuinderbos genotype had higher production in treatment II, while in the other genotypes production was equal among treatments or even higher for isolated prothalli. Interaction between genotype and treatment was significant for *A. scolopendrium *(Table 3). Figure 2 suggests that this interaction might also be present for *P. aculeatum*, but this relation was not significant in week 30 (but was significant in week 16; Wald *χ*^2 ^= 0.990, *P *= 0.019). For *P. setiferum *the interaction between genotype and treatment was significant (Table 3), but no longer when the genotype from outside the Kuinderbos was excluded (Wald *χ*^2 ^= 0.114, *P *= 0.735), which was the only genotype with increased production at treatment II (Figure 2).

## Discussion

Breeding and population genetic studies on various fern species have shown that inbreeding depression is often reduced in allopolyploids compared with their diploid parents, because recessive deleterious or lethal alleles are less likely to be expressed after genome duplication [19,21,38]. The same pattern could be expected for our study species, among which are two tetraploids (*P. aculeatum *and *A. trichomanes *subsp. *quadrivalens*) and two diploids (*A. scolopendrium *and *P. setiferum*). Indeed, previous studies on their mating systems were in line with the expectations [29,39]. However, the results of our experiments show a very different pattern.

Two key results can be extracted from our data. First of all, intraspecific variation in selfing rate was present in all species, and two species seem to show variation in mating strategy among genotypes sampled from the Kuinderbos. Secondly, Kuinderbos genotypes of all species showed surprisingly high capacities for sporophyte production via intragametophytic selfing. As a result, the predicted differences in mating strategy between diploid and tetraploid species were not observed. Strong selection for selfing genotypes following long-distance dispersal, a mechanism previously described as Baker's law [13], may explain these observations. Below, we discuss why our results may indicate that Baker's law also applies to ferns.

### Intraspecific variation in gametophyte isolate potential and mating strategy

In breeding studies very similar to ours, Klekowski [17,20] and various others interpreted the percentage of bisexual gametophytes that failed to produce a sporophyte as a measure of the genetic load in the parental sporophyte from which spores were derived for the study. Based on this principle, intraspecific variation in the percentage of isolated gametophytes producing a sporophyte may be interpreted in terms of differences in genetic load between the parental genotypes [e.g. [40]]. This principle is, however, less applicable for completely homozygous parental sporophytes, since in this case all gametophytic genotypes are identical and lethal recessive alleles should cause the death of all zygotes produced by any of the sib gametophytes (although it should be noted that part of the genetic load may consist of alleles that are deleterious but not lethal, and that the expression of such alleles may still reduce the success of sporophyte formation [41]). As genetic analysis suggested that most parental sporophytes were homozygous, differences in the percentage of sporophyte-bearing isolated gametophytes per parental genotype may primarily have been due to other factors than genetic load. As we were interested in the overall potential of a certain parental genotype to produce sporophytes rather than in the effect of genetic load alone, we transplanted gametophytes in the pre-sexual stage. A failure to produce a sporophyte in isolation may thus also have been caused by a failure to reach a functional bisexual status or by a gametophytic morphology that limits the transport of gametes on the gametophyte [16]. Taking this into consideration, we consistently apply the terminology of Peck et al. [18] and refer to the success of sporophyte production in treatment I as 'gametophyte isolate potential' instead of 'selfing potential' (the latter being defined as the success of sporophyte production on bisexual gametophytes [18]). Both gametophyte morphology and sexual status are influenced by environmental factors, but also by genetic factors [16,42].

As gametophytes were cultured in a randomized setting under controlled conditions, species and genotypic differences in fertilization success within treatments were not likely due to environmental variation. However, the presence of a second gametophyte in treatment II might have resulted in slightly altered conditions [26]. Most importantly, paired gametophytes may affect each other's sexual status by excreting antheridiogens, which inhibit further growth of nearby gametophytes and stimulate them to become male e.g. [43]. However, our observation that nearly all gametophytes had a normal, heart-like shape and produced female reproductive organs suggests a limited effect of antheridiogens. Humidity was nearly 100% in all petri dishes and a surplus of nutrients was added to the culture medium. We therefore assume that a difference between the success of sporophyte production in isolation and when paired with a genetically different gametophyte (that is, the treatment effect) has a genetic basis. Enhanced production in treatment II may either be due to genetic load or a gametophyte morphology or gametangial ontogeny that inhibits self-fertilization. Differences between parental genotypes in this respect (genotype-treatment interaction) are therefore interpreted in terms of genetically based differences in mating strategy.

Our results show clear intraspecific variation in gametophyte isolate potential in all four species. Moreover, all species, except *A. trichomanes *subsp. *quadrivalens*, showed differences between parental genotypes in the relative success of sporophyte production among treatments: one genotype showed a clearly enhanced sporophyte production in the presence of a second genotype (suggesting a preferentially outcrossing mating system), while the other genotypes did not show this effect.

As genetic differences may influence fertilization success in many different ways, the presence of genotypic variation in selfing rates is not unlikely. Only a very limited number of studies has, however, previously tested the mating system of multiple genotypes of the same fern species in a single experiment [but see [18,29,30]]. Our results reconfirm the intraspecific variation in mating system shown for *A. scolopendrium *by Wubs et al. [30] and show similar variation in three additional species. As all previous breeding studies that used multiple genotypes per species indeed found genotypic variation in mating system, we stress the importance of testing a range of different genotypes before drawing any conclusions on the dominant mating system of a fern species. In some previous studies e.g. [19] spores from multiple populations were pooled to infer a general mating strategy per species. This may, however, not be very informative on the actual mating systems the species may show in nature. We note that even if a certain mating strategy is most abundant among the genotypes of a species, a particular mating strategy may be overrepresented in habitats which impose a selective pressure on capacities for a certain type of mating (e.g., disjunct habitats; see below).

### Did selection for selfing genotypes obscure variation in selfing capacity among ploidy levels?

We showed a clear potential for fertilization and sporophyte production via intragametophytic selfing for all investigated Kuinderbos genotypes of each of the four species. Gametophyte isolate potential was high for all genotypes. This high potential to produce viable sporophytes on a single gametophyte implies that most gametophytes do become bisexual, that self-fertilization is successful in the presence of water, and that early inbreeding depression (i.e. mortality of inbred sporophytic embryos due to expression of genetic load [44]) is very limited in these genotypes. Moreover, allowing the option of intergametophytic crossing by providing a genetically different mate (treatment II) rarely increased sporophyte production compared with isolated cultures for the Kuinderbos genotypes. As intragametophytic selfing was most likely also common in treatment II, the absence of a treatment effect suggests that Kuinderbos genotypes not only have a high capacity to self-fertilize, but also an overall selfing strategy. A replacement of self-fertilization by cross-fertilization can, however, not be excluded without genetic analysis of the ± 250 sporophytes produced in paired cultures. Unfortunately, we were unable to perform such an analysis within this study. Apomixis is common in ferns [45], but is unlikely to explain the high gametophyte isolate potential shown for our study species. All gametophytes developed archegonia and sporophytes were always attached to the gametophyte close to the location of its archegonia. In most apomictic ferns, sporophytes are formed elsewhere on the prothallus [45].

The high selfing capacity observed for both *Asplenium trichomanes *subsp. *quadrivalens *and *P. aculeatum *is in line with results of previous studies [29,39]. However, for *Asplenium trichomanes *subsp. *quadrivalens *the selfing capacity of the Kuinderbos genotypes was much higher than that of the Swiss genotypes tested by Suter et al. [29]. The mainland genotype "Eck" used in our study showed a gametophyte isolate potential that was similar to that of the Swiss genotypes (< 50%). The Swiss populations sampled by Suter et al. [29] were located in mountainous regions that harbour numerous populations of this subspecies and where gene flow thus might be relatively high. Our "Eck" genotype was sampled near Maastricht (the Netherlands), also a region where this subspecies is relatively common. Genotypic variation in selfing capacity thus seems present across the species' European distribution, but selfing rates were consistently higher for genotypes from the most isolated populations. This is in line with the pattern predicted based on the hypothesis of selection for selfing genotypes after long-distance dispersal.

Similar, but more surprising results were found for *P. setiferum*. Pangua et al. [39] reported isolated gametophytes derived from a Spanish population of this species to be totally incapable of intragametophytic selfing, and therefore described the diploid species *P. setiferum *as an obligate out crosser. However, our results show that a genotype from outside the Kuinderbos was capable of intragametophytic selfing and that genotypes from Kuinderbos populations established by long-distance dispersal showed even higher capacities to self-fertilize, and no difference in sporophyte production between paired and isolated cultures. First of all, this shows that a selfing strategy is present even in the diploid *P. setiferum*. Secondly, the difference in mating strategy between the Iberian genotypes, located close to the centre of the species geographic distribution [39], and the Kuinderbos genotypes, obtained from isolated populations, is again in line with predictions based on selection for selfing following long-distance dispersal.

A clear capacity for self-fertilization was also observed in the other diploid species, *Asplenium scolopendrium*. All three Kuinderbos genotypes showed very high sporophyte production in isolation. A previous study by Wubs et al. [30] also showed selfing capacity for 8 out of 9 parental genotypes tested. The fact that Kuinderbos genotypes of both diploid species are clearly capable of self-fertilization and that, at least for the Kuinderbos, no significant difference in gametophyte isolate potential was found between the diploid and polyploid species is in marked contract with previous studies showing increased selfing in polyploid ferns e.g. [21]. This might imply that the effect of polyploidization on the selfing capacity of fern gametophytes is less straightforward than is sometimes assumed [e.g. [40]]. It may however also be a result of the specific characteristics of the Kuinderbos populations: isolated populations, which were likely established through single-spore colonization following long-distance dispersal. Selection for selfing genotypes (i.e. Baker's law [13]) may have obscured any differences in the dominant mating system of the species studied. We predict that the two diploid species might show a larger proportion of obligatory outcrossing genotypes on a European scale, but that strong mate limitation upon arrival in the isolated Kuinderbos has largely prevented the establishment of genotypes incapable of intragametophytic selfing.

As we studied only a limited number of Kuinderbos genotypes per species, we cannot exclude that some of the successful colonizers in this forest were obligate outcrossers which did find a nearby mate. Results of a population genetic study for the same four species (G.A. de Groot, unpublished data) suggested that population establishment by intergametophytic crossing has at least occurred once for *P. setiferum*. Previous results by Wubs et al. [30] also suggest a lack of selfing capacity for a few Kuinderbos genotypes of *A. scolopendrium*. Interestingly, the only Kuinderbos genotype of *A. scolopendrium *which shows enhanced sporophyte production in treatment II of our study (plant AS3) was also the only one that was heterozygous (and thus must have resulted from cross-fertilization).

## Conclusions

Two important conclusions can be drawn from our data. First, we show that even within diploid fern taxa previously reported to be clear outcrossers, some genotypes may be very well able to self-fertilize. We show intraspecific variation in mating system for four different species and predict that the presence of such variation may be common, at least among temperate calcicolous ferns.

Secondly, gametophyte isolate potential [18] was consistently high for all genotypes obtained from the Kuinderbos, across all four species tested, despite differences in ploidy level. A selective pressure on selfing capacity imposed by strong mate limitation upon spore arrival (i.e. Baker's law [13]) may have obscured any differences in dominant mating system present at a larger scale between the diploid and polyploid species studied. The occurrence of such a selection effect among ferns has been suggested before based on observations of mating-system variation among populations e.g. [22,46,47].

Although numbers of investigated genotypes were limited, our results support the idea that selfing ability is of great importance for fern population establishment after long-distance dispersal [30,48] and that the advantages of single spore establishment favour selfing genotypes during long-distance colonization in ferns. This is in line with results for other plant groups and helps to explain the evolution of inbreeding in fern species [24,41].

## Competing interests

The authors declare that they have no competing interests.

## Authors' contributions

GAG, ERJW and HJD conceived and designed the study, BV and GAG performed the experiment and collected the data, GAG and ERJW performed the data analysis. GAG, HJD, ERJW and RHJE drafted the manuscript. All authors read and approved the final manuscript.

## Supplementary Material

Additional file 1 Genotypes of parent sporophytes used to obtain spores for the experiment. Genotypes (rows) are based on four polymorphic microsatellite loci per species (columns), and differ between all used plants. Different alleles are designated by different letters. Certain heterozygotes (diploids with two alleles or tetraploids with three or four alleles) are given in bold face. Plant codes as in Table 1. Codes of microsatellite loci follow De Groot et al. 35. Click here for file

Additional file 2 **Genotypes of parent sporophytes used to obtain spores for the experiment**. Genotypes (rows) are based on four polymorphic microsatellite loci per species (columns), and differ between all used plants. Different alleles are designated by different letters. Certain heterozygotes (diploids with two alleles or tetraploids with three or four alleles) are given in bold face. Plant codes as in Table 1. Codes of microsatellite loci follow De Groot et al. [35]. Click here for file

## References

[B1] Cain ML Milligan BG Strand AE Long-distance seed dispersal in plant populations American Journal of Botany 2000 87 1217 1227 10.2307/2656714 10991892

[B2] Hampe A Plants on the move: The role of seed dispersal and initial population establishment for climate-driven range expansions Acta Oecologica 2011 in press

[B3] Svenning J-C Condit R Biodiversity in a warmer world Science 2008 322 206 207 10.1126/science.1164542 18845738

[B4] Thomas CD Bodsworth EJ Wilson RJ Simmons AD Davies ZG Musche M Conradt L Ecological and evolutionary processes at expanding range margins Nature 2001 2001 411 577 581 10.1038/35079066 11385570

[B5] Darling E Samis KE Eckert CG Increased seed dispersal potential towards geographic range limits in a Pacific coast dune plant New Phytologist 2008 178 424 435 10.1111/j.1469-8137.2007.02349.x 18194144

[B6] Hanski I Eralahti C Kankare M Ovaskainen O Siren H Variation in migration propensity among individuals maintained by landscape structure Ecology Letters 2004 7 958 966 10.1111/j.1461-0248.2004.00654.x

[B7] Arnaud-Haond S Teixeira S Massa S Billot C Saenger P Coupland G Duarte CM Serrão EA Genetic structure at range-edge: low diversity and high inbreeding in SE Asia mangrove (*Avicennia marina*) populations Molecular Ecology 2006 15 3515 3525 10.1111/j.1365-294X.2006.02997.x 17032254

[B8] Hoffmann AA Blows MW Species borders: ecological and evolutionary perspectives Trends in Ecology and Evolution 1994 9 223 227 10.1016/0169-5347(94)90248-8 21236827

[B9] Lloyd DG Self- and cross-fertilization in plants. II. The selection of self-fertilization International Journal of Plant Sciences 1992 153 370 380 10.1086/297041

[B10] Nathan R Levin SA Dispersal biogeography Encyclopaedia of biodiversity 2001 2 San Diego: Academic Press 127 152

[B11] Baker HG Self-compatibility and establishment after "long-distance" dispersal Evolution 1955 9 347 348 10.2307/2405656

[B12] Baker HG Support for Baker's law-as a rule Evolution 1967 21 853 856 10.2307/2406780 28563079

[B13] Stebbins GL Self-fertilization and population variability in the higher plants American Naturalist 1957 91 337 354 10.1086/281999

[B14] Jain SK The evolution of inbreeding in plants Annual Review of Ecology and Systematics 1976 7 469 495 10.1146/annurev.es.07.110176.002345

[B15] Dassler CL Farrar DR Significance of gametophyte form in long-distance colonization by tropical, epiphytic ferns Brittonia 2001 53 352 369 10.1007/BF02812705

[B16] Raghavan V Developmental biology of fern gametophytes 1989 Cambridge University Press, Cambridge

[B17] Klekowski EJ Genetic load in *Osmunda regalis *populations American Journal of Botany 1973 60 146 154 10.2307/2441101

[B18] Peck JH Peck CJ Farrar DR Influences of life history attributes on formation of local and distant fern populations American Fern Journal 1990 80 126 142 10.2307/1547200

[B19] Flinn KM Reproductive biology of three fern species may contribute to differential colonization success in post-agricultural forests American Journal of Botany 2006 93 1289 1294 10.3732/ajb.93.9.1289 21642193

[B20] Klekowski EJ Dyer AF The genetics and reproductive biology of ferns The experimental biology of ferns 1979 London: Academic Press 133 170

[B21] Masuyama S Watano Y Trends for inbreeding in polyploid pteridophytes Plant Species Biology 1990 5 13 17 10.1111/j.1442-1984.1990.tb00188.x

[B22] Klekowski EJ Evidence against genetic self-incompatibility in the homosporous fern *Pteridium aquilinum* Evolution 1972 26 66 73 10.2307/2406983 28555766

[B23] Klekowski EJ Populational and genetic studies of a homosporous fern, *Osmunda regalis* American Journal of Botany 1970 57 1122 1138 10.2307/2441278

[B24] Soltis PS Soltis DE Evolution of inbreeding and outcrossing in ferns and fern-allies Plant Species Biology 1990 5 1 11 10.1111/j.1442-1984.1990.tb00187.x

[B25] Soltis DE Soltis PS The distribution of selfing rates in homosporous ferns American Journal of Botany 1992 79 97 100 10.2307/2445202

[B26] Korpelainen H Intragametophytic selfing does not reduce reproduction in *Dryopteris filix-mas* Sexual Plant Reproduction 1996 9 117 122 10.1007/BF02153059

[B27] Soltis DE Soltis PS Breeding system of the fern *Dryopteris expansa*: evidence for mixed mating American Journal of Botany 1987 74 504 509 10.2307/2443829

[B28] Soltis PS Soltis DE Genetic variation and population structure in the fern B*lechnum spicant *(Blechnaceae) from western North America American Journal of Botany 1988 75 37 44 10.2307/2443903

[B29] Suter M Schneller JJ Vogel JC Investigations into the genetic variation, population structure, and breeding systems of the fern *Asplenium trichomanes *subsp. *quadrivalens* International Journal of Plant Sciences 2000 161 233 244 10.1086/314258 10777447

[B30] Wubs ERJ De Groot GA During HJ Vogel JC Grundmann M Bremer P Schneider H Mixed mating system in the fern *Asplenium scolopendrium: *implications for colonization potential Annals of Botany 2010 106 583 590 10.1093/aob/mcq157 20682575 PMC2944980

[B31] Ranker TA Genetic diversity, mating systems, and interpopulation gene flow in neotropical *Hemionitis palmata *L. (Adiantaceae) Heredity 1992 69 175 183 10.1038/hdy.1992.111

[B32] Pryor KV Young JE Rumsey FJ Edwards KJ Bruford MW Rogers HJ Diversity, genetic structure and evidence of outcrossing in British populations of the rock fern *Adiantum capillus-veneris *using microsatellites Molecular Ecology 2001 10 1881 1894 10.1046/j.1365-294X.2001.01343.x 11555233

[B33] Bremer P The ferns of the Kuinderbos (the Netherlands), the establishment of 23 species in a planted forest Acta Botanica Neerlandica 1980 29 351 357

[B34] Bremer P The colonization of a former sea-floor by ferns PhD thesis 2007 Wageningen University

[B35] De Groot GA Korpelainen H Wubs ERJ Erkens RHJ Isolation of polymorphic microsatellite markers and tests of cross-amplification in four widespread European calcicole fern species American Journal of Botany 2011 98 e319 e322 10.3732/ajb.1100051 22012927

[B36] Haufler CH Homospory 2002: An odyssey of progress in pteridophyte genetics and evolutionary biology BioScience 2002 52 1081 1093 10.1641/0006-3568(2002)052[1081:HAOOPI]2.0.CO;2

[B37] Klekowski EJ Reproductive biology of the Pteridophytes. III. A study of the Blechnaceae Botanical Journal of the Linnean Society 1969 62 361 377 10.1111/j.1095-8339.1969.tb01973.x

[B38] Hedrick PW Genetic load and the mating system in homosporous ferns Evolution 1987 41 1282 1289 10.2307/2409093 28563604

[B39] Pangua E Quintanilla LG Sancho A Pajarón S A comparative study of the gametophytic generation in the *Polystichum aculeatum *group (Pteridophyta) International Journal of Plant Sciences 2003 164 295 303 10.1086/346165

[B40] Ranker TA Geiger JMO Ranker TA, Haufler CH Population genetics Biology and Evolution of Ferns and Lycophytes 2008 Cambridge: Cambridge University Press 107 133

[B41] Lande R Schemske DW The evolution of self-fertilization and inbreeding depression in plants. I. Genetic models Evolution 1985 39 24 40 10.2307/2408514 28563655

[B42] Ranker TA Houston HA Is gametophyte sexuality in the laboratory a good predictor of sexuality in nature American Fern Journal 2002 92 112 118 10.1640/0002-8444(2002)092[0112:IGSITL]2.0.CO;2

[B43] Schneller JJ Haufler CH Ranker TA Antheridiogen and natural gametophyte populations American Fern Journal 1990 80 143 152 10.2307/1547202

[B44] Husband BC Schemske D Evolution of the magnitude and timing of inbreeding depression in plants Evolution 1996 50 54 70 10.2307/2410780 28568860

[B45] Walker RG Dyer AF The cytogenetics of ferns The experimental biology of ferns 1979 London: Academic Press 88 132

[B46] Cousens MI Gametophyte ontogeny, sex expression, and genetic load as measures of population divergence in *Blechnum spicant* American Journal of Botany 1979 66 116 132 10.2307/2442514

[B47] Crist K Farrar D Genetic load and long distance dispersal in *Asplenium platyneuron* Canadian Journal of Botany 1983 61 1809 1814 10.1139/b83-190

[B48] Lott MS Volin JC Pemberton RW Austin DF The reproductive biology of the invasive ferns *Lygopodium microphyllum *and *L. japonicum (Schizaeaceae)*: implications for invasive potential American Journal of Botany 2003 90 1144 1152 10.3732/ajb.90.8.1144 21659214

