# Supplementary material for: Inter-and intraspecific variation in fern mating systems after long-distance colonization: the importance of selfing
Source: BMC Plant Biol. 2012 Jan 4;12:3. doi: 10.1186/1471-2229-12-3 (PMC3305374; doi:10.1186/1471-2229-12-3)
Supplement: Additional file 1 — Genotypes of parent sporophytes used to obtain spores for the experiment. Genotypes (rows) are based on four polymorphic microsatellite loci per species (columns), and differ between all used plants. Different alleles are designated by different letters. Certain heterozygotes (diploids with two alleles or tetraploids with three or four alleles) are given in bold face. Plant codes as in Table 1. Codes of microsatellite loci follow De Groot et al. 35. [file 1471-2229-12-3-S1.PDF]

Additional file 1 - Genotypes of parent sporophytes used to obtain spores for the experiment.

Genotypes (rows) are based on four polymorphic microsatellite loci per species (columns), and differ between all used plants. Different alleles are designated by different letters. Certain heterozygotes (diploids with two alleles or tetraploids with three or four alleles) are given in bold face. Plant codes as in Table 1. Codes of microsatellite loci follow De Groot *et al.* (2011).

a: *Asplenium scolopendrium* (ASPS, 2n)

Plant	Microsatellite loci			
	AS-7	***AS-9***	***AT-5***	***AT-10***
AS1	BB	AA	AA	AA
AS2	AA	AA	AA	AA
AS3	**BC**	AA	**AB**	AA
RC	**BD**	AA	AA	AA

b: *Asplenium trichomanes* ssp. *quadrivalens* (ASPT, 4n)

Plant	Microsatellite loci			
	AT-1	***AT-3***	***AT-8b***	***AT-12***
AT1	BBBB	BBBB	BBBB	CCCC
AT2	CCCC	BBBB	AD??	BBBB
Eck	AAAA	AB??	CCCC	AC??
B	BBBB	BBBB	AD??	AAAA

c: *Polystichum setiferum* (POLS, 2n)

Plant	Microsatellite loci			
	PS-3	***PS-5***	***PA-3***	***PA-6***
PS1	BB	CC	AA	BB
PS2	BB	AA	AA	BB
BRS	**AB**	**AB**	AA	**AB**

d: *Polystichum aculeatum* (POLA, 4n)

Plant	Microsatellite loci			
	PA-4	***PA-5b***	***PA-6***	***PS-5***
PA1	AAAA	AB??	AB??	BBBB
PA2	AAAA	BBBB	BBBB	AAAA
PA3	AAAA	DE??	BBBB	BBBB
SG	AC??	DDDD	BBBB	BC??
BRA	BC??	CCCC	BBBB	AB??
