# Supplementary figures and images for: Inter-and intraspecific variation in fern mating systems after long-distance colonization: the importance of selfing
Source: BMC Plant Biol. 2012 Jan 4;12:3. doi: 10.1186/1471-2229-12-3 (PMC3305374; doi:10.1186/1471-2229-12-3)

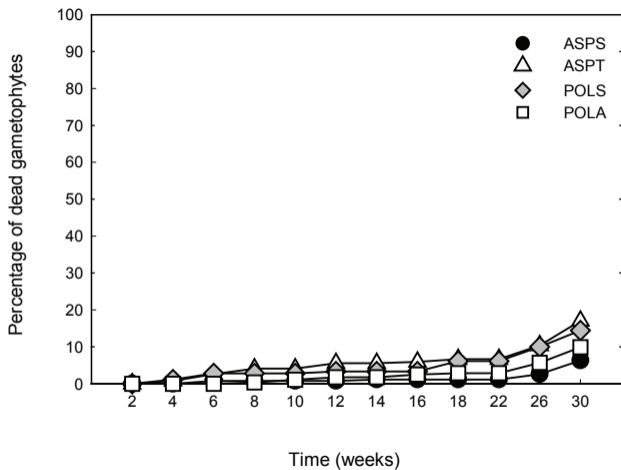

Supplement: Additional file 2 — Genotypes of parent sporophytes used to obtain spores for the experiment. Genotypes (rows) are based on four polymorphic microsatellite loci per species (columns), and differ between all used plants. Different alleles are designated by different letters. Certain heterozygotes (diploids with two alleles or tetraploids with three or four alleles) are given in bold face. Plant codes as in Table 1. Codes of microsatellite loci follow De Groot et al. [35]. [file 1471-2229-12-3-S2.PDF]
